# Highly protein-loaded melt extrudates produced by small-scale ram and twin-screw extrusion - evaluation of extrusion process design on protein stability by experimental and numerical approaches

**DOI:** 10.1016/j.ijpx.2023.100196

**Published:** 2023-07-01

**Authors:** Katharina Dauer, Kevin Kayser, Felix Ellwanger, Achim Overbeck, Arno Kwade, Heike P. Karbstein, Karl G. Wagner

**Affiliations:** a Department of Pharmaceutics, Institute of Pharmacy, University of Bonn, Bonn, Germany; b Institute of Process Engineering in Life Sciences, Food Process Engineering, Karlsruhe Institute of Technology, Karlsruhe, Germany; c Technische Universität Braunschweig, Institute for Particle Technology (iPAT) and Center of Pharmaceutical Engineering, Braunschweig, Germany

**Keywords:** Hot-melt extrusion, Protein formulation, Solid-state stability, Computational fluid dynamics, Numerical simulation

## Abstract

Understanding of generation, extent and location of thermomechanical stress in small-scale (< 3 g) ram and twin-screw melt-extrusion is crucial for mechanistic correlations to the stability of protein particles (lysozyme and BSA) in PEG-matrices. The aim of the study was to apply and correlate experimental and numerical approaches (1D and 3D) for the evaluation of extrusion process design on protein stability. The simulation of thermomechanical stress during extrusion raised the expectation of protein degradation and protein particle grinding during extrusion, especially when TSE was used. This was confirmed by experimental data on protein stability. Ram extrusion had the lowest impact on protein unfolding temperatures, whereas TSE showed significantly reduced unfolding temperatures, especially in combination with kneading elements containing screws. In TSE, the mechanical stress in the screws always exceeded the shear stress in the die, while mechanical stress within ram extrusion was generated in the die, only. As both extruder designs revealed homogeneously distributed protein particles over the cross section of the extrudates for all protein-loads (20–60%), the dispersive power of TSE revealed not to be decisive. Consequently, the ram extruder would be favored for the production of stable protein-loaded extrudates in small scale.

## Highlights

• Better process understanding through combination of experimental and simulation data.
• 1D/3D simulation valuable tool for evaluation of ram and TS extrusion process design.
• 1D simulation identifies critical process steps in TSE.
• 3D simulation boosts mechanistic understanding and identifies critical process areas.
• 60% protein-load protective effect due to less decreased unfolding temperatures.

## Graphical abstract

**Figure:**
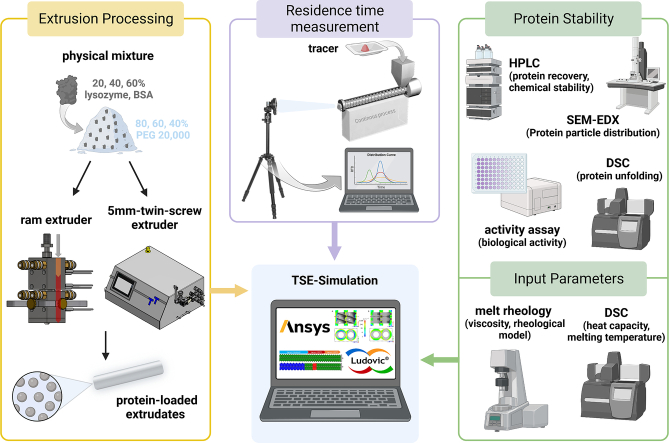

## 1 Introduction

The biopharmaceutical sector has grown rapidly over the last two decades (Martins et al., 2022). However, compared to small-molecule based drug products, biopharmaceuticals are more complex and have to be parenterally administered mostly as liquid formulation (Antosova et al., 2009). One of the challenges is that many protein-based drugs are not stable in liquid formulations. Therefore the solid-state is preferred for a wide range of used protein-based drugs for biopharmaceutical development (Lai and Topp, 1999; Pikal, 2009). Physical and chemical stability of protein-based drugs is considered as bottleneck for a successful biopharmaceutical development. Even in the solid state, protein degradation can occur during the whole life cycle of a protein formulation (Manning et al., 2010; Pikal, 2009). As the thermal stability of a protein depends on the relation between the degree of protein degradation and process-related stress applied the unfolding behavior of a protein is commonly studied.

Lyophilization and spray-drying are the mainly used techniques to convert biopharmaceutical formulations from an aqueous to a solid-state formulation providing improved stability during storage (Horn et al., 2018; Massant, 2020; Pansare and Patel, 2019; Schüle et al., 2007; Sharma et al., 2021).

In pharmaceutical industry, hot melt extrusion (HME) is mainly used as continuous and robust manufacturing technology for the production of solid dosage forms (Crowley et al., 2007). In the last few years, the application of HME was expanded to biopharmaceutics and can be used for the production of long-term release systems for parenteral administration (e.g., protein-loaded implants) or for solid-state protein stabilization by embedding proteins in polymeric carriers (Ghalanbor et al., 2010, Ghalanbor et al., 2012; Vollrath et al., 2017).

In early formulation development, 9- or 12-mm co-rotating twin-screw extruders (TSE) are frequently used for the production of amorphous solid dispersions (ASD) mainly resulting in a solubility enhancement of poorly water-soluble drugs, but still require batch sizes of about 20–30 g, which result in substantial amounts of drug substance and development costs, respectively (Jiang et al., 2023; Zecevic and Wagner, 2013). The amount of protein-based drug candidates within early formulation development studies however, is often limited and thus small-scale HME is particularly relevant (Dauer et al., 2021). Small-scale HME such as ram extrusion and TSE ideally enables the processing of batch sizes below 3 g accompanied with high yields of the produced protein-loaded extrudate under short processing times of <3 min (Dauer et al., 2022). The aim is a homogenous embedding of protein powder particles in a polymeric matrix by HME without negatively affecting the protein stability. The embedding of protein drugs in polymeric or lipid-based matrices by HME approaches at a small-scale level has been described in previously published works. Ghalanbor et al. assessed the feasibility of HME for preparing lysozyme-PLGA-implants and showed a complete recovery of active lysozyme from PLGA implants (Ghalanbor et al., 2010), whereas Cossé et al. used 5- and 9-mm mini-scale twin screw extruders for the preparation of BSA-PLGA-implants. A special focus was paid to the erosion properties and the in vitro release of the embedded BSA (Cossé et al., 2017). Another working group introduced the production of solid lipid implants (SLI) containing 10 to 20% either of a lyophilized mAb or a f_ab_-fragment by small-scale TSE (5 mm) at 35 °C and 40 rpm. The analytical investigations revealed a process-related impact on the physical protein stability as there were a loss in the monomer content of both the mAb and the f_ab_-fragment and changes in secondary structure elements of the mAb (Vollrath et al., 2017). The use of novel, biodegradable phase-separated poly(ε-caprolactone-PEG)-*block*-poly(*ε*-caprolactone), [PCL-PEG]-*b*-[PCL]) multiblock copolymers with different block ratios and with a low melting temperature (49 to 55 °C) for the production of protein-loaded implants was successfully shown by Stanković et al. The spray-dried protein powders goserelin, insulin, lysozyme, carbonic anhydrase, and albumin were incorporated into the polymers, whereby all proteins completely preserved their structural integrity as determined after extraction of the proteins from the polymeric implants (Stanković et al., 2015; Stanković et al., 2013).

The works published so far are limited only to the production of protein-loaded extrudates by small-scale HME and characterization of the implants in terms of protein stability, protein recovery, and protein release. A mechanistic understanding and investigation of how process parameters such as type of extruder, feed-rate, residence time distribution, and process-related stress factors (thermomechanical stress profiles along the ram extruder or TSE barrel, or in the extruder die) affect protein stability, remain “black boxes”. Apart from that, the use of computational simulations to gain better HME process understanding and correlation of the simulation data with experimental results of protein stability was hitherto not considered.

One major issue in small-scale extrusion, especially when 5-mm TSE are used, has been identified to be a long residence time of up to 5 min, which subsequently results in an increased thermomechanical stress and a potentially negative impact on protein stability (Cossé et al., 2017; Patil et al., 2016). It is therefore crucial to carefully balance the process parameters: (i) Feed rate, (ii) screw design, (iii) screw speed, (iv) die geometry, and (v) L/D-ratio, in order to avoid spots of elevated shear and/or thermal stress along the process. As melt temperature and pressure in small-scale extrusion is usually determined in the die only, information on the extent and location of above-mentioned hot spots along the process are easily missed. The gap towards a mechanistic understanding of the interactions between process variables and quality attributes needs therefore to be filled via numeric simulation (Bochmann et al., 2018; Emin et al., 2021; Emin and Schuchmann, 2013a; Zecevic and Wagner, 2013). The present study evaluates ram extrusion and TSE as small-scale extrusion designs to produce protein-loaded extrudates. During ram extrusion, a powder mixture is loaded into a heated barrel where a piston is forced down onto the molten material (Dauer et al., 2022). The main shear stress generated during ram extrusion is found as the reservoir of the ram extruder is tapered into the die section. On the contrary, a TSE provides the opportunity of dispersive and distributive mixing (Wilson et al., 2012; Zecevic et al., 2018). The powdered starting material is fed into the feed zone and transported to the subsequent zones by the turning motion of the screw along the barrel under pre-heating (Bravo et al., 2000). This process of conveying introduces mixing and heat into the material through both external heaters and viscous heat dissipation (Crowley et al., 2007). In the die zone head pressure is developed, which is determined by several factors: (i) the molten blend viscosity, (ii) the flow rate of the molten blend, and (iii) the die temperature (Gedde et al., 2021). TSE provides a continuous system with much better mixing, shorter residence times, ease of material feeding, high kneading (distributive) and dispersion capacities as injection molding or ram extrusion (Wilson et al., 2012).

For our study, we used the hydrophilic polymer polyethylene glycol (PEG) 20,000 and the two model proteins lysozyme and bovine serum albumin (BSA) for the production of protein-loaded extrudates by ram extrusion and TSE. PEG 20,000 was selected as challenging polymer, since it exhibits a complex crystallization and melting behavior (Paberit et al., 2020) and thus a narrow extrusion process window. Due to the low melting temperature of PEG 20,000, it enables extrusion at a temperature of below 65 °C, which in combination with a short residence time can minimize the risk of heat-induced protein degradation during HME processing. PEG 20,000 is waxy and shows a very high viscosity below the melting temperature (not extrudable due to an extensive torque) and a very low viscosity above the melting temperature (not extrudable due to liquification). Additionally, the screw configuration including conveying screws, and screws with a single 90° kneading element as well as the screw speed influence the resulting mechanical stress on polymer and protein particles (Emin and Schuchmann, 2013b). The present work aimed to include relevant extrusion process parameters such as: (i) screw configuration, (ii) screw speed, and (iii) residence time distributions (RTD) to facilitate potential correlations of the parameters with experimental data on polymer, and protein characteristics: (i) rheology, (ii) melting temperature shifts, (iii) protein recovery rates, and (iv) biological activity). Comparison of extrusion experiments were evaluated with the computations of the 1D and 3D simulation software Ludovic® and Ansys Polyflow®, respectively. The goal was to enable a fundamental and early starting point for the production of protein-loaded extrudates with sufficient protein stability and prior protein formulation development of long-term release systems by small-scale HME processing.

## 2 Materials and methods

### 2.1 Materials

Lysozyme from chicken egg-white (Cat. No. L6876) was obtained from AppliChem GmbH (Darmstadt, Germany). Polyethylene glycol (PEG) 20,000 was obtained from Carl Roth (Karlsruhe, Germany). Bovine serum albumin (BSA) was purchased from Merck KGaA (Darmstadt, Germany). All chemicals were of analytical grade or equivalent purity.

### 2.2 Preparation of physical mixtures and protein-loaded extrudates by small-scale ram and twin-screw extrusion (5 mm)

PEG 20,000 flakes were milled in 3 cycles of 30 s utilizing a high-shear mixer at a fixed shear rate (Krups Mixette type 210, Krups, Frankfurt am Main, Germany) and passed through a 650 μm sieve to remove larger particles. For the preparation of protein-loaded extrudates, a physical mixture composed of polymer and 20, 40, or 60% *w*/w either of lysozyme or BSA powder was blended for 10 min at 50 rpm using a turbula mixer (Willy A. Bachofen AG, Muttenz, Switzerland). The physical mixtures of PEG 20,000 and protein powder (Table 1) were either ram extruded or hot melt extruded by using twin-screw extrusion (TSE).

**Table 1:** Overview of the compositions and prepared protein-loaded extrudates by ram or twin-screw extrusion.

Formulation	Protein	Components (%)		Preparation method
		Protein	PEG 20,000	
LR20	Lysozyme	20	80	Ram extrusion
LR40	Lysozyme	40	60	Ram extrusion
LR60	Lysozyme	60	40	Ram extrusion
BR20	BSA	20	80	Ram extrusion
BR40	BSA	40	60	Ram extrusion
BR60	BSA	60	40	Ram extrusion
LC20	Lysozyme	20	80	TSE (conveying)
LC40	Lysozyme	40	60	TSE (conveying)
LC60	Lysozyme	60	40	TSE (conveying)
BC20	BSA	20	80	TSE (conveying)
BC40	BSA	40	60	TSE (conveying)
BC60	BSA	60	40	TSE (conveying)
LK20	Lysozyme	20	80	TSE (kneading)
LK40	Lysozyme	40	60	TSE (kneading)
LK60	Lysozyme	60	40	TSE (kneading)
BK20	BSA	20	80	TSE (kneading)
BK40	BSA	40	60	TSE (kneading)
BK60	BSA	60	40	TSE (kneading)

A self-built ram extruder was used for the preparation of protein-loaded extrudates with lower shear stress as previously described by (Dauer et al., 2022). A barrel of 10 cm length and an inner hole of 10 mm diameter consisted of three heating zones and was equipped with a cylindrical die (diameter 1 mm, length 7 mm). Extrudates were prepared by feeding 3 g of the physical mixture into the 10-mm hole of the ram extruder. The temperature of the first segment (filling-zone) was set to 58 °C, and the other two segments were set to 63 °C. The mixture was molten for three minutes and the blends were then extruded through the die by a driving piston with a speed of 1 mm/s.

**Fig. 1:**
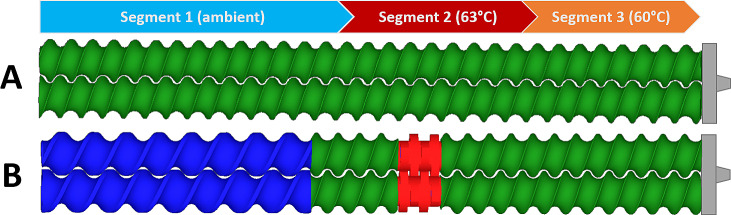
Screw configurations of the 5 mm twin-screw extruder with: **A** only conveying elements; **B** a single kneading element; 5 mm pitch (green), 7.5 mm pitch (blue), 90° kneading element (red); Segment 1 (ambient temperature), segment 2 (63 °C), and segment 3 (60 °C). (For interpretation of the references to color in this figure legend, the reader is referred to the web version of this article.)

TSE was performed using a 5 mm co-rotating twin-screw extruder (ZE 5, Three-Tec GmbH, Seon, Switzerland) with a length to diameter ratio (L/D) of 15:1 and two heating zones. The outlet of the extruder was equipped with a 1 mm die and either a conveying screw configuration, or a screw configuration with a single kneading element were used, which are presented in Fig. 1. The physical mixtures were fed into the extruder by using a powder belt conveyor (GUF-P Mini AD / 475 / 75, mk Technology Group, Troisdorf, Germany) at constantly kept feed-rates of 0.4, and 0.8, or 2.0 g/min ± 5% for lysozyme- and BSA-composed mixtures (Table 1), respectively. The screw speed was set to 100, 150, or 200 rpm. Extrudates were collected in the steady-state of the extrusion process and the amount of extruded formulations was 1.5 to 3.0 g depending on the feed-rates and process parameters.

### 2.3 Residence time distribution (RTD)

For TSE trials a 5 mm twin-screw extruder (ZE 5, Three-Tec GmbH, Seon, Switzerland) was used with two different screw configurations (Fig. 1). PEG 20,000 powder was extruded at 60 and 63 °C at five different feed-rates of 0.4, 0.6, 1.0, 1.4, or 2.0 g/min. The screw speed was set to 100 or 200 rpm. In order to investigate a potential effect of protein concentration on residence time, MRT measurements of powder blends composed of PEG 20,000 and 0, 20, 40, or 60% protein at a fixed feed-rate of 0.6 g/min were also performed. Mean residence times (MRTs) were measured with iron oxide as dye tracer (Sicovit® Red 30 E 172, BASF SE, Ludwigshafen, Germany) and calculated by using a camera system (ExtruVis3, ExtruVis, Riedstadt, Germany).

### 2.4 Particle size distribution analysis of protein powder

Protein powder were analyzed in terms of particle size distribution and particle shape via dynamic image analysis (DIA) (Camsizer® X2, Retsch Technology, Haan, Germany). The continuous minimum diameter (*d*_*xc*_ *min*) of the analyzed particles was used for the particle size determination (*n* = 4).

### 2.5 Mechanical single crystal analysis

For mechanical analysis, single crystal compression tests were performed using a TiboIndenter Hysitron (Ti900, Bruker Minneapolis MN, USA) equipped with a 100 μm diameter diamond flatpunch probe. The protein crystals were dispersed on a flat sapphire substrate so that the average distance between them was >200 μm to ensure individual loading. BSA crystals were sieved with a 125 μm mesh sieve prior to sample preparation.

The size of each crystal was determined prior to each loading with the calibrated instrument optics by determining the longest axis of the project surface and the length approximately perpendicular to it. The load function was displacement controlled to a maximum of 10 μm for Lysozyme and 20 μm for BSA crystals at a displacement rate of 1 μm s^−1^. Compression tests were performed in ambient air at a temperature of 22 °C and humidity between 45 and 60%. After each compression test, the flat punch probe was cleaned of any crystal debris. The force-displacement curves obtained in this way were analyzed for fracture events, which appear as discontinuities with a clear drop in force. The breaking force and breaking displacement were read from the first breaking event in each case.

### 2.6 Scanning electron microscopy coupled with energy-dispersive X-ray spectroscopy (SEM-EDX)

A scanning electron microscope (SU 3500, Hitachi High Technologies, Krefeld, Germany), equipped with a backscattered electron detector (BSE) was used to investigate the morphology of the surfaces and cross sections of the prepared extrudates. BSE images were collected at an acceleration voltage of 5 kV at a variable pressure mode of 30 Pa. The cut extrudates were placed on an aluminum stub. Samples were sputtered with a thin platin layer (Sputter Coater, Cressington Scientific Instruments, Watford, England). Protein particle distribution was examined by elemental mapping of the cross sections of extrudates for the characteristic X-ray peak of nitrogen. The elemental distributions were investigated by SEM combined with an energy-dispersive X-ray detector (EDX) (EDAX Element-C2B, Ametek, Weiterstadt, Germany). The percentage of detected nitrogen was evaluated by the TEAM software (Version 4.4.1, Ametek, Weiterstadt, Germany).

### 2.7 Determination of protein concentration and recovery rate by RP-HPLC

Protein concentration in extrudates was determined by HPLC (LC20AT Solvent Delivery Pump, CBM-40lite system controller, SIL-10AF Autosampler, CTO-10A column oven, Shimadzu, Kyoto, Japan) using a C18 reversed phase column (NUCLEOSIL 120–3, C18, 5 μm, 125 × 4 mm, MACHEREY-NAGEL GmbH & Co. KG, Düren, Germany). Approximately 50 mg of the extrudates were accurately weighed and dissolved in Milli-Q® water (*n* = 3). Samples vials were cooled at 5 °C in the autosampler and 10 μL of each sample was injected. The solvent system consisted of water/acetonitrile/trifluoroacetic acid (A: 100/0/0.1, B: 0/100/0.1, *V*/V). A linear gradient method was applied (0–5-7 min 5–95-5%B) at a flow rate of 1 mL/min for 7 min and a column temperature of 30 °C.

Chromatograms were obtained using an UV-detector (SPD-40 UV Detector, Shimadzu, Japan) at 220 nm.

### 2.8 Vacuum compression molding (VCM)

Samples for rheological and DSC measurements were prepared with a VCM tool (MeltPrep GmbH, Graz, Austria) as described by (Treffer et al., 2015). In brief, a setup with a diameter of 20 mm (500 mg) and 5 mm (20 mg) for rheological and DSC measurements was used, respectively. PEG 20,000 and mixtures were molded at 65 °C for 5 min.

### 2.9 Melt rheology

An oscillatory, small amplitude (SAOS) rheometer (HAAKE MARS III, Thermo Scientific, Karlsruhe, Germany) equipped with a roughened plate-plate geometry (d = 20 mm) was used. All experiments were conducted in controlled deformation AutoStrain (CDAuto-Strain) mode after equilibration of the samples for 5 min at the starting temperature with a gap height of 1.2 mm. PEG 20,000 and PEG 20,000-BSA mixtures containing 20, 40, or 60% *w*/w BSA were measured using the following parameters: amplitude 0.0018% at 1.0–10.0 Hz for the temperatures 62.8, 63.5, 64.0, 64.5 and 65.0 °C. The amplitude of each blend and plain PEG 20,000 was determined to be in the linear viscoelastic range (LVR) by amplitudes sweeps. A horizontal time-temperature superposition (TTS) was conducted where feasible resulting in master curves shifted to 64.0 °C (Hajikarimi and Moghadas Nejad, 2021). The resulting master curve(s) were fitted to the power law fit:

(1) $$\eta^{*}=K∙\gamma^{n}$$

where *K* is the consistency Index (Pa∙s), γ is the shear rate (s^−1^) and *n* is the power law index. Subsequently, to allow simulations an expression of temperature dependency is necessary. Therefore, temperature sweeps with an underlying heating rate of 0.2 °C/min were conducted to allow the determination of the Arrhenius activation energy of flow (*E*_*A*_) employing the following Eqs. (2), (3):

(2) $$|\eta^{*}|=A∙e^{\frac{-E_{A}}{R*T}}$$

(3) $$\ln|\eta^{*}|=A-\frac{E_{A}}{R}∙\frac{1}{T}$$

where |η*| is the measured complex viscosity (Pa∙s), *A* is the pre-exponential factor, *R* is the gas constant (8.314 J∙K^−1^∙mol^−1^), *T* is the respective temperature (K) and *E*_*A*_ is the slope of the resulting plot.

### 2.10 Differential scanning calorimetry (DSC)

DSC studies of PEG 20,000, protein powder, and protein-loaded extrudates were performed with a DSC 2 (Mettler Toledo, Gießen, Germany) equipped with an auto sampler, nitrogen cooling and nitrogen as purge gas (30 mL/min). The system was calibrated with indium and zinc standards. Extrudates were milled with a mortar and pestle. At least three samples of ∼10 mg were accurately weighed in 40 μL aluminum crucibles with a pierced lid. DSC scans were recorded from 25 °C to 230 °C using a heating rate of 10 °C/min. STAR^e^ software (Mettler Toledo, Gießen, Germany) was employed for acquiring thermograms. Thermograms were normalized for sample weight. The heat capacity (input parameter for simulation) of PEG 20,000, pure BSA and Lysozyme, and protein-PEG 20,000 mixtures were determined using a multi-frequency temperature modulation (TOPEM® mode) with an underlying heating rate of 2 °C/min, a pulse height of 1 °C, from 25 °C to 100 °C and a constant nitrogen purge (30 mL/min).

### 2.11 Biological activity of lysozyme after extrusion

Lysozyme activity was determined by a fluorescence-based assay (EnzChek® Lysozyme Assay Kit, Molecular Probes Europe BV, Leiden, The Netherlands) using a suspension of *Micrococcus lysodeikticus* labeled with fluorescein. The assay determines the lysozyme activity on the cell walls of *Micrococcus lysodeikticus*, which are labeled to such a degree that fluorescence is quenched. The fluorescence increase was measured using a microplate reader with a fluorescein filter and OptiPlate™-96 F microwell plates (VICTOR3™ Multilabel Plate Reader; 96-Well plates black, PerkinElmer Life and Analytical Sciences, Shelton, Germany). Preparation of DQ Lysozyme substrate stock suspension, lysozyme standard curve, as well as the procedure were conducted according to the manufacturer's protocol. The reaction mixtures were incubated at 37 °C for 30 min, protected from light. The fluorescence intensity of each reaction in a microwell plate was measured at 494 nm (absorption maximum) and 518 nm (emission maximum). The fluorescence values obtained from the control without enzyme were subtracted.

### 2.12 Simulation of mean residence time

The one-dimensional (1D) simulation software Ludovic® V6.0 PharmaEdition (Sciences Computers Consultants, Saint Etienne, France) was used for computing thermo-mechanical analysis. This approach allows the calculation of various parameters along the screw profile (e.g., temperature, residence time, etc.). The model assumes non-isothermal flow conditions and an instantaneous melting prior to the first restrictive screw element. Due to the unknown filling ratio of a starve-fed extruder, the computation starts at the die and proceeds backwards in an iterative procedure until the final product temperature is achieved. Input parameters were: (i) screw configuration, (ii) screw speed, (iii) temperatures of the segments, (iv) feed-rate, (v) thermal characteristics of the extruded mixture (i.e., heat capacity, density, thermal conductivity: 0.16 W/m∙K, melting temperature, and melt rheology data).

### 2.13 3D isothermal simulation of the die sections and the screw section

The calculation of the numerical equations was performed with the commercial software Ansys Polyflow® by Ansys Inc. (Canonsburg, PA, USA). The software is specifically suitable for HME processing and provides a finite element method solver for highly viscous media. Simulations were performed on a cluster server, computing one node with 32 Intel Xeon Gold 6230 processors and 70 GB of Random-Acess Memory.

The geometric dimensions of the simulated dies correspond to the dimensions of the dies of the ram extruder and TSE used in experiments. To reduce the computational effort, the geometries were quartered and calculated with symmetry planes. A mesh independence study resulted in computational meshes with 162,000 elements for the ram extruder die and 347,850 elements for the TSE die. A three-dimensional overview of the geometries and according meshes can be found in the Supplementary Material (Fig. S1-S3). The boundary conditions for the ram extruder were chosen as 1 mm/s normal velocity at the inflow, outflow conditions at the outlet, 1 mm/s normal velocity at the reservoir wall, zero velocity condition at the conical die inlet wall and the die wall. The boundary conditions for the TSE die were chosen as 0.15 g/min mass flow rate inlet at the inflow, outflow conditions at the outlet and zero velocity at all walls.

In order to simulate the rotating screw elements of the TSE, the mesh superposition technique was used (Avalosse, 1996). A detailed description of the application of the technique used on TSE can be found in a recent work (Emin et al., 2021). The geometric dimensions of the simulated screw elements correspond to the conveying element (5 mm pitch) and the 90° kneading element shown in Fig. 1. A mesh independence study resulted in computational meshes with 245,000 elements for the barrel, 206,984 elements for each conveying element, and 237,711 elements for each kneading element. A three-dimensional overview of the geometries and according meshes can be found in the Supplementary Material (Fig. S1-S3). The boundary conditions were chosen as 0.6 g/min mass flow rate inlet at the inflow, outflow conditions at the outlet, zero velocity at the outer barrel walls, screw elements and inner barrel walls rotate with 200 rpm. While wall slip can occur at high shear rates in the extrusion process, it was neglected for the simulations for simplicity. Accordingly, the no slip condition was assumed at the surface of the barrel and the rotating screw. Due to its symmetry, only half a revolution of the screw elements was simulated in 60-time steps.

Energy equation and gravity acceleration were neglected. Linear velocities and linear pressure were chosen for interpolation settings. Iterations on the viscosity were performed with a Picard scheme. The material parameters used for the simulations correspond to the 100% PEG batch of the extrusion trials. The density of the material was set as 1200 kg/m^3^. The viscosity was described using a power law fit according to section 2.9. The fitted model is given in eq. (4):

(4) $$\eta \left(\dot{\gamma }\right)=2,887,070∙{\dot{\gamma }}^{-0.6783}\;\mathrm{Pa}∙s$$

To further analyze the simulation results, particle tracking analysis were performed. Therefore, 2000 mass- and volume less, non-interacting particles were randomly distributed at the inflow. Based on the velocity field solved, particles move through the domain where each point of the trajectories and its corresponding values were tracked and recorded.

### 2.14 Statistical analysis

Statistical analysis and testing for statistical significance were carried out using Prism (GraphPad Software Inc., La Jolla, USA).

## 3 Results

### 3.1 Protein content in extrudates and protein particle distribution over the cross section of extrudates

**Fig. 2:**
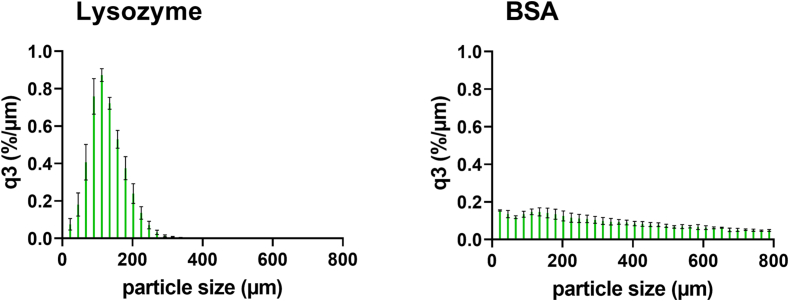
Particle size distribution of protein powder (lysozyme and BSA); q3 is the cumulative distribution referred to the percentage (%) of particles below that micron size (μm); error bars represent the standard deviation of four measurements by DIA.

The particle size distribution analysis of the used protein powder particles for the preparation of highly-loaded protein-extrudates showed a narrow particle size distribution for lysozyme (*d*_*50*_ = 133 ± 7 μm), whereas BSA (*d*_*50*_ = 444 ± 70 μm) showed a very broad particle size distribution (Fig. 2).

**Fig. 3:**
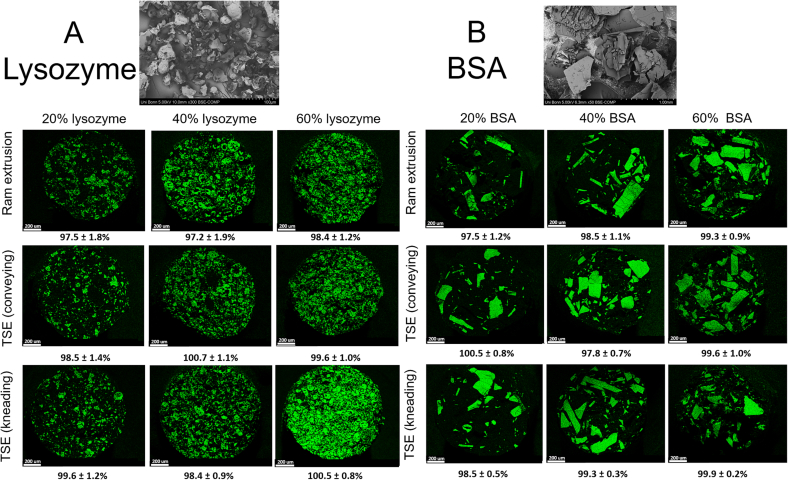
SEM-images of the used protein powder; protein particle distribution by SEM-EDX and elemental mapping of nitrogen (green spots) on cross-section of 20, 40, and 60% protein-loaded extrudates (**A** lysozyme and **B** BSA) prepared by ram or twin-screw extrusion (screw speed: 150 rpm) at 63 °C and protein recovery rates determined by RP-HPLC (*n* = 3) below the SEM-EDX-images. The scale bar of SEM-images corresponds to 200 μm. (For interpretation of the references to color in this figure legend, the reader is referred to the web version of this article.)

The degree of protein powder particle embedding in the polymeric matrix and protein particle distribution over the cross section of extrudates produced by ram extrusion or TSE was analyzed by SEM-EDX (Fig. 3). The surface of the extrudates prepared by TSE appeared smoother compared to extrudates produced by ram extrusion. The cross-section cut of the extrudates prepared by ram extrusion showed no pores or cracks inside of the extrudates, whereas the extrudates prepared by TSE showed only few pores. Elemental mapping of the cross-sections showed an overall homogeneous distribution of protein particles in extrudates prepared by ram extrusion and TSE. Furthermore, the protein particles were only dispersed in the PEG- matrix and were not dissolved or notably grinded during extrusion. The protein recovery rate determined by HPLC was over 97% for all extruded samples.

### 3.2 Mechanical single crystal analysis

**Fig. 4:**
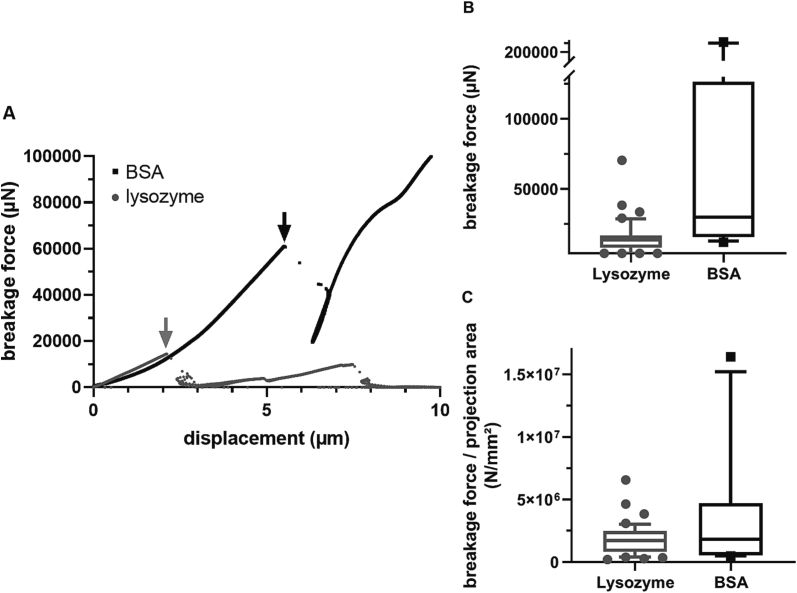
**A** Representative force - displacement data for the compression of lysozyme (gray) and BSA (black) crystals, each with a significant breakage event (arrows). Box plot representation of **B** breakage force and **C** breakage force/ projection area for lysozyme (gray) and BSA (black); horizontal line in the center of the box indicates the median, whiskers are drawn to the 90th and 10th percentiles of the data set.

The mechanical properties of the protein crystals were determined using micro compression experiments. Fig. 4A shows a representative force-displacement curve for lysozyme (gray) and BSA with a significant fracture event. For lysozyme, 46 crystals were examined, 44 of which showed one or more breakage events. For BSA, 28 crystals were tested, of which 13 showed an evaluable breakage event. If more than one fracture event occurred, only the first one was evaluated, as the number and size of the particles for the following loading is unknown.

Fig. 4B and C summarizes the micro-compression results. The values for breakage force and breakage displacement were significantly lower for lysozyme than for BSA. However, BSA with an average particle size of 94 μm was also coarser than lysozyme with an average particle size of 52 μm for the crystals examined by micro-compression. However, the fracture force for BSA was twice that of lysozyme in relation to the projected area. This ratio, which is similar to a fracture stress, tends to be a size independent material parameter. For comparison, compression tests of native lysozyme crystals in mother liquid are mentioned by (Cornehl et al., 2014), who determined a mean bursting force of only 238.3 ± 124.5 μN for a comparable particle size (48.7 ± 1.6 μm), which corresponds to a fracture stress of about 3.2 ∙ 10^4^ N/mm^2^. Therefore, the dried crystals used in this work are comparatively stable.

### 3.3 Unfolding temperature

**Fig. 5:**
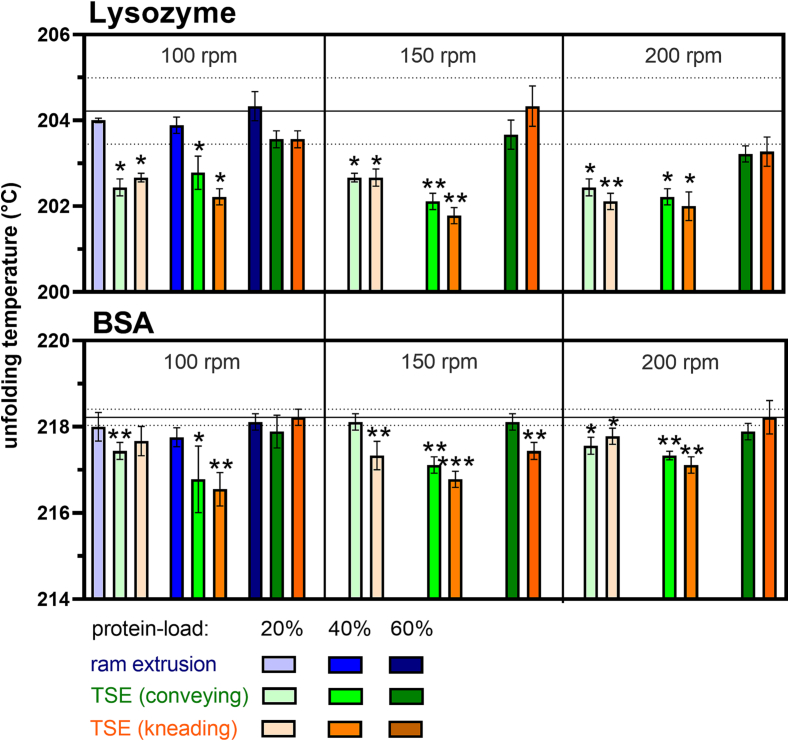
Unfolding temperatures of 20, 40, and 60% protein-loaded (lysozyme and BSA) extrudates prepared by ram extrusion (blue) or TSE with only conveying screw configuration (green), or screws containing a single 90° kneading element (orange) at 100, 150, or 200 rpm screw speed, at 63 °C; error bars represent the standard deviation of three measurements for the unfolding temperature by DSC; the line shows the melting temperature of the unprocessed protein (reference) and the dotted lines represent the standard deviation (n = 3); statistical significance is compared to the reference and depicted by asterisks (*): * *p* < 0.05, ** *p* < 0.01, *** *p* < 0.001. (For interpretation of the references to color in this figure legend, the reader is referred to the web version of this article.)

Unfolding temperatures of lysozyme and BSA in protein-loaded extrudates produced by ram extrusion and TSE were determined by DSC (Fig. 5). The reported unfolding temperatures of the protein powders served as references and were compared to the produced protein-loaded extrudates. Lysozyme- and BSA-loaded extrudates (20, 40, and 60% protein-load) prepared by ram extrusion showed no significantly reduced unfolding temperatures compared to the unprocessed neat protein powder. Processing of 40% lysozyme by TSE at 100, 150, and 200 rpm screw speed and at a constant feed-rate of 0.4 g/min shifted the unfolding temperature towards lower temperatures whereas the unfolding temperatures of 60% lysozyme-loaded extrudates were not significantly reduced and were independent of the applied screw speed. The unfolding temperatures of 60% BSA-loaded extrudates were not reduced neither when produced by ram extrusion nor by TSE. 40% BSA-loaded extrudates prepared by TSE with conveying or kneading screw configuration showed significantly reduced unfolding temperatures.

### 3.4 Lysozyme activity after extrusion

**Fig. 6:**
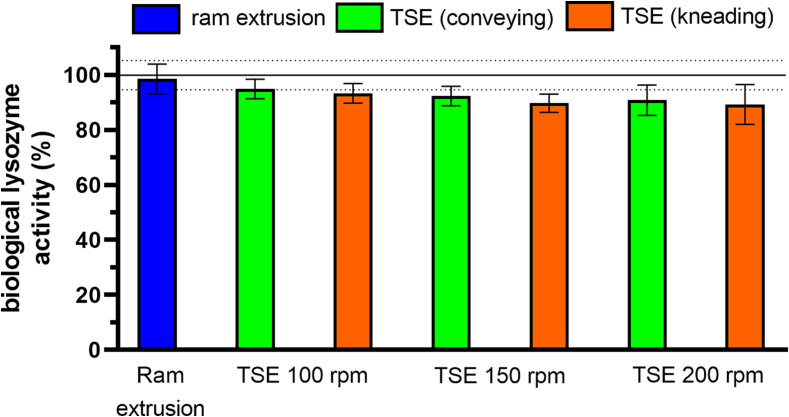
Biological activity of lysozyme of 40% lysozyme-loaded extrudates prepared by ram extrusion (blue) or TSE with only conveying screw configuration (green), or screws containing a single 90° kneading element (orange), at 63 °C and 100, 150, and 200 rpm screw speed; samples were immediately analyzed after extrusion processing; error bars represent the standard deviation of three measurements; the line shows the biological activity of the unprocessed lysozyme (reference) and the dotted lines represent the standard deviation (n = 3). (For interpretation of the references to color in this figure legend, the reader is referred to the web version of this article.)

The biological activity of lysozyme in 40% lysozyme-loaded extrudates was investigated immediately after ram extrusion and TSE with conveying and kneading screw configuration, at 100, 150, and 200 rpm and at 63 °C. The activity of lysozyme embedded in melt extrudates by ram extrusion or TSE was higher than 90% and thus maintained the full biological activity when compared to the activity of the unprocessed lysozyme powder, which was 99.86 ± 5.29% (*t*-test: *p* < 0.05) (Fig. 6).

### 3.5 Residence time distribution

In order to receive information about the residence time distribution of the material within the extrusion barrel in dependence of feed-rate, screw configuration, and screw speed, MRT measurements were performed.

**Fig. 7:**
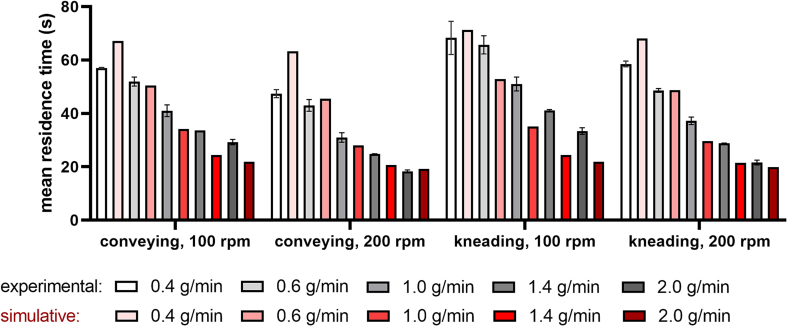
Mean residence time as a function of screw configuration (conveying, kneading; screw speed: 100 and 200 rpm); 100% PEG 20,000 was fed into the extruder at five different feed-rates (0.4, 0.6, 1.0, 1.4, and 2.0 g/min); error bars represent the standard deviation of three MRT measurements.

Fig. 7 shows the determined MRT by an experimental and simulative approach for five feed-rates, two screw configurations and varying screw speed. For the simulation of MRT by Ludovic®, melt rheology data were key input parameters (refer to Supplementary Material, Figs. S4–6). The experimental MRT for a feed-rate of 0.4 g/min was found at 57.0 ± 0.2 s at 100 rpm and 47.4 ± 1.7 s at 200 rpm, with a comparably broad distribution using the conveying screw configuration, whereas simulation revealed MRTs of 67.1 s at 100 rpm and 63.3 s at 200 rpm. The experimental MRT measurements using the screw configuration with a single kneading element at a feed-rate of 0.4 g/min resulted in 68.3 ± 6.2 s at 100 rpm and 58.5 ± 1.1 s at 200 rpm. The simulated MRTs at a feed-rate of 0.4 g/min were found at 71.3 s and 68.1 s at 100 rpm and 200 rpm, respectively.

MRTs for 2.0 g/min at 100 and 200 rpm were found at 29.3 ± 1.0 s and 18.3 ± 0.5 s for the conveying screw configuration, and 33.4 ± 1.2 s and 21.6 ± 0.9 s for the kneading screw configuration, respectively. The MRTs obtained by simulation were 21.9 s and 19.2 s for the conveying screw configuration at 100 and 200 rpm, respectively, whereas the MRT data for the kneading screws were 21.9 s at 100 rpm and 19.8 s at 200 rpm. Higher feed-rates resulted in more narrow residence time distributions (data not shown). The simulation accuracy of MRTs was higher at increasing feed-rates. For feed rates of 1.0 g/min and higher the simulated MRTs were within the experimental MRTs including their standard deviations. The smallest deviations between experimental and simulated MRT were found for the conveying and kneading screw configuration at a screw speed of 200 rpm.

**Fig. 8:**
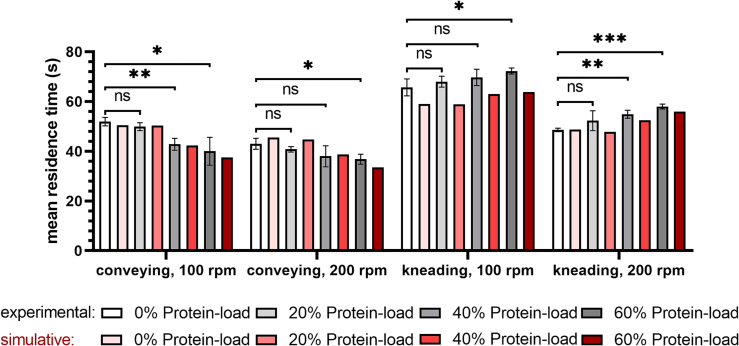
Mean residence time as a function of screw configuration (conveying, kneading; screw speed: 100 and 200 rpm) and protein-load (0, 20, 40, and 60% protein-PEG 20,000 mixtures) at a feed-rate of 0.6 g/min; simulated MRT is displayed in gray color; experimental MRT is displayed in red color; error bars represent the standard deviation of three MRT measurements; unpaired *t*-test (two-sample assuming equal variances) was used and statistical significance was depicted by asterisks (*) for MRT. ns = not significant, significant differences are indicated by asterisks: *, p < 0.05, **, p < 0.01 and, ***, p < 0.001. (For interpretation of the references to color in this figure legend, the reader is referred to the web version of this article.)

Fig. 8 shows the MRT data obtained by experiments or simulations as a function of protein concentration at a fixed feed-rate of 0.6 g/min. For the conveying screw configuration, the MRT decreased with increasing protein concentration as 0% compared to 60% protein-load showed a decrease in the experimental and simulated MRT of 11.9 s at 100 rpm and 6.2 s at 200 rpm and 12.9 s at 100 rpm and 12.0 s at 200 rpm, respectively. For the screw configuration containing a single kneading element, the MRT was slightly increased with increasing protein concentration. For the mixture containing 60% protein MRT was prolonged compared to 0% protein load about 6.6 s at 100 rpm and 9.4 s at 200 rpm, while simulation of the MRTs at 100 and 200 rpm revealed an increase of 4.8 s and 7.3 s, respectively.

### 3.6 Mechanical stress history in extrusion processing

**Fig. 9:**
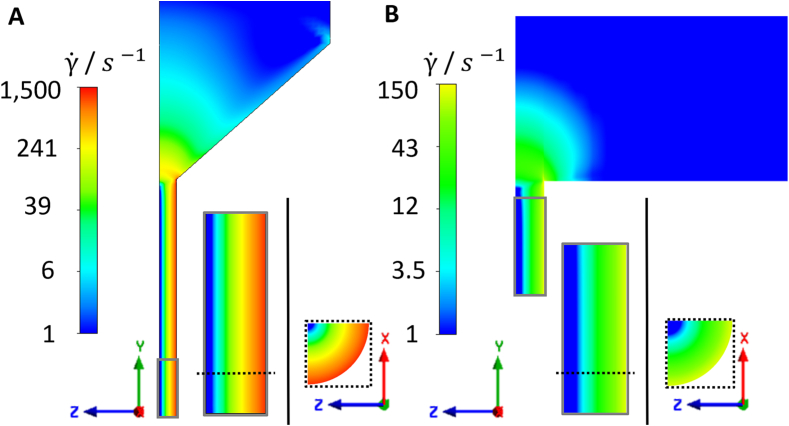
Color plots of the shear rate distribution of **A** ram extruder die; **B** twin-screw extruder die.

Fig. 9 shows color plots of the shear rate distribution in the dies of the ram extruder and the TSE. Low shear rates were found in the reservoir of the ram extruder. As the capillary narrows in the direction of flow, the flow is accelerated, resulting in increasing shear rates. In the area of the smallest radius, shear rates up to 1500 s^−1^ were observed. In this area the shear rate increased over the radius of the die and reached its maximum at the wall. Likewise, the highest shear rate was also achieved in the area of the smallest radius for the TSE with values up to 150 s^−1^. The shear rate profile is similar to the ram extruder, but with values 10-times lower.

**Fig. 10:**
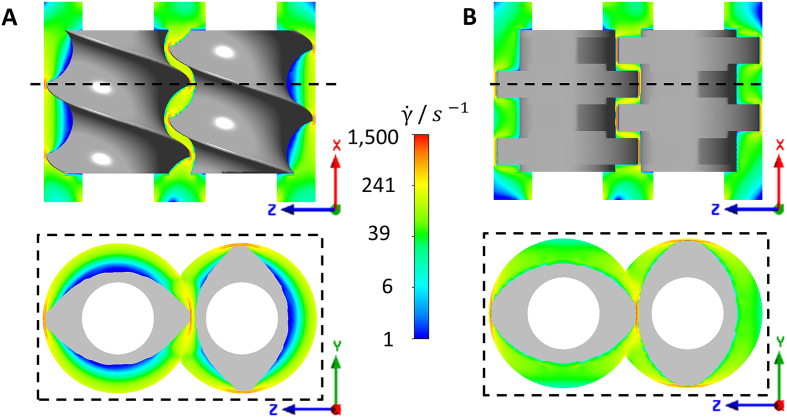
Color plots of the shear rate distribution of **A** TSE conveying elements (5 mm pitch) die; **B** TSE 90° kneading elements.

The shear rate distribution of a fully filled screw section of the TSE is shown for a pair of conveying elements and kneading elements in Fig. 10. For both type of elements, the highest shear rates were observed in the gap between the screw tip and the barrel wall as well as in the gap between the rotating elements. The maximum shear rates were similar for both element types with values up to 1500 s^−1^. For the kneading element, the shear rates were higher than 30 s^−1^ in the whole area, whereas for the conveying elements in the channel also low shear rates were found.

**Fig. 11:**
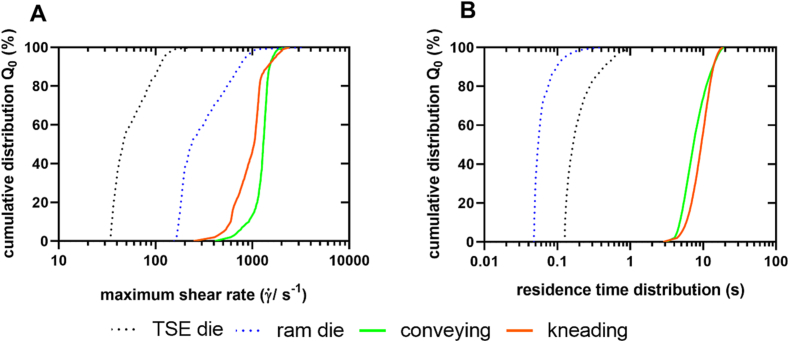
**A** Cumulative number distribution of the maximum shear rate along the particles tracked for the TSE die, the ram die, the conveying elements and the kneading elements; **B** Residence time distribution along the particles tracked for the twin-screw extruder die (area of smallest radius only), the ram extruder die (area of smallest radius only), the conveying elements and the kneading elements.

Although the color plots of the different geometries provided information about the shear rate distribution, it was not possible to conclude about the mechanical stress experienced by the material in these sections. Therefore, particle tracking analyses were performed with the solved velocity fields and particle trajectories were obtained. The maximum shear rate and residence time were calculated for each trajectory and cumulative distributions were created. Fig. 11 shows the cumulative distributions of the maximum experienced shear rate and the residence time distribution for the different dies and screw elements. As expected, the material experienced higher shear rates in the screw section than in the die section for both screw configurations and in comparison to the shear stress in the ram extruder die. The median (Q_0,50_) was found at 237 s^−1^ for the ram extruder die, 46 s^−1^ for the TSE die, 1295 s^−1^ for the conveying elements, and 1040 s^−1^ for the kneading elements.

Regarding the residence time distribution, it can be observed that particles remained longer in the die of the TSE than in the ram extruder die. However, the residence times were shorter than in the simulated screw elements. Comparing the screw elements, a slightly longer residence time of the particles in the kneading block were observed, although the volume which passed the kneading block was similar and the mass flow constant, respectively. The median (Q_0,50_) was found at 0.05 s for the ram die, 0.16 s for the TSE die, 7.4 s for the conveying elements, and 9.4 s for the kneading elements.

## 4 Discussion

While for the ram extruder melting and extruding, i.e., introducing mechanical stress, were applied sequentially, these processes were introduced simultaneously during TSE and thermomechanical stress was certainly present for the unmolten and molten material during TSE processing. Monitoring and characterization of the magnitude and duration of the generated thermomechanical stress is often not feasible and highlights the need of computational simulation for gaining insights into small-scale HME processing including temperature profiles, mechanical stress distributions, and RTDs. The particle tracking approach utilized describes infinitesimally small, massless particles moving through the resolved flow fields. Accordingly, the motion of actual protein particles was only partially replicated.

In regard of mechanical stress, the die area of both extruders was comparable and the highest shear rates were found close to the wall, gradually decreasing towards the center of the die channel. Particles dispersed in the PEG-melt passing the die rather in the center, subsequently experienced a lower shear stress compared to those closer to the wall. For TSE the highest shear rates were present in the screw section and particularly in the gap between the screws and the gap between screw and barrel. The increased shear rate for the kneading element can be attributed to different flow profiles resulting from the restriction of 90° kneading discs.

The MRTs of the blends in the heated barrels were short, namely 3 min and <80 s, for ram extrusion and TSE, respectively, and thus did not lead to a dissolution of protein particles but rather a solely dispersing of protein particles in the PEG-matrix. In this study, lysozyme and BSA particles could be regarded as fillers during extrusion, since the particle size of the used protein powders was identical for the starting material and particles determined in the cross section of extrudates. Ram extrusion facilitated sufficient dispersion of protein particles for protein-loads in the range of 20 to 60%. Consequently, the distributive mixing power of TSE was not necessary for the production of protein-loaded extrudates and TSE was thus not superior in particle distribution compared to ram extrusion. The simulation of maximum shear rates raised the expectation of particle size reduction during extrusion, especially when TSE was used. Micro compression analysis showed that BSA crystals were more ductile and less fragile than solid lysozyme particles (Fig. 4). However, the mechanical stress generated during ram extrusion and TSE was not high enough to break either the lysozyme or the BSA particles. Since the protein particles showed also an elastic deformation behavior, a compensation of local shear stress peaks during extrusion could be possible (Kubiak et al., 2021; Suzuki et al., 2018). However, a correlation of the simulated mechanical stress with the micro compression analysis could not be established in this study, as more advanced approaches would be needed, such as coupling CFD simulations with DEM simulations (Frungieri et al., 2020).

Ram extrusion had the lowest impact on unfolding temperatures as the main mechanical stress was generated only in the die for <1 s as confirmed by the results of 3D simulation. The ram extrusion process had unexceptionally no negative effect on the unfolding temperatures of lysozyme and BSA in the various extrudates compared to the unprocessed proteins and were also independent of the protein-load. As experimental and simulated data revealed, ram extrusion provides a gentle approach for the production of protein-loaded extrudates.

In contrast, a protein-load effect on the unfolding temperature was observed for extrudates produced by TSE. Interestingly a higher protein-load of 60% was protective resulting in a less pronounced decrease of unfolding temperatures. According to a correlation of the unfolding temperatures with the simulated temperature profiles along the TSE process and MRTs, we hypothesize that the generated shear stress during TSE were distributed to a larger protein particle collective and thus, protein-loads higher than 50% should be favored for the embedding of a thermally stable protein. For a better understanding of TSE processing, MRTs determined by TSE experiments and 1D simulation (Ludovic®) were compared. The experimental MRTs for the pure PEG and 20% protein-polymer mixture were congruent with simulated data (Fig. 7). In mixtures with 40% or 60% protein, the impact on melt rheology behavior (Figs. S4–6) and thus the MRT is no longer neglectable and was reliably predicted by Ludovic®. The MRT in the kneading element was slightly increased, implying that protein particles were entrapped within the gaps of the kneading element, resulting in an increased viscous dissipation (see Fig. S7). This is confirmed by the results of unfolding temperatures and provides an important correlation for the evaluation of extrusion process design on protein stability. The shorter simulated MRTs for the screws comprising the kneading element at 100 rpm might resulted in a deviation between the apparent melt viscosity in the experiment compared to the anticipated melt viscosity of 1D simulation (Fig. 8). As the simulation anticipates a molten continuum to begin with, in reality this condition needs to be established within the first part of the extrusion process. 1D simulation revealed that the highest temperature of the molten blend was achieved by entering in the die zone (Fig. S7). At 100 rpm the contribution of the introduced viscous dissipation to the overall melt energy was lower compared to 200 rpm (peak temperatures: 75.6 °C and 78.1 °C, respectively). As the kneading elements have no conveying functionality the mass will pile up in front of the element. As the condition of being molten in reality would have been reached later at 100 rpm, this was likely the reason of the more pronounced deviation of experimental and simulated MRTs using segmented screws at low screw speed.

Compared to the reliably simulated thermomechanical and temperature profiles and RTDs along the TSE barrel and the die and as torque could not adequately measured in the 5 mm TSE experiments, 1D simulation could not reveal pressure distribution, which would otherwise be standard. For an increased simulation accuracy, improved instrumentation especially for pressure and melt temperatures along the entire process length would be necessary. Especially, the pressure would indeed be an excellent variable to validate the 3D simulations of the die section. Advanced instrumentation would facilitate improved model validation as pressure and temperature profiles could be correlated. Unfortunately, such advanced instrumentation was not available for the applied extruders and will be part of further studies. Additionally, some simplifications during simulation might have added to an higher variance, such as isothermal conditions, no wall slippage and no viscosity transition from waxy condition into melt. Subsequently, the simulated pressure values are likely to be higher than in an experiment. Therefore, simulations were primarily intended to compare geometries and to improve process understanding for the appropriate selection of protein-related process and formulation demands.

## 5 Conclusion

A fundamental and early starting point for the production of highly protein-loaded extrudates with sufficient protein stability and enabling an understanding of extrusion processing prior protein formulation development of potential long-term release systems by small-scale extrusion processing was provided. A mechanistic understanding and investigation of how process parameters such as type of extruder, feed-rate, RTD, and process-related stress factors (thermomechanical stress profiles along the ram extruder or TSE barrel, or in the extruder die) affect protein stability, was hitherto not considered in the literature. In our study, several extrusion process characteristics and material properties such as heat capacity, density, melt rheology data of the investigated polymer-protein mixtures have been considered as input parameters for 1D and 3D simulations. The combination of experimental and numerical approaches resulted in a better understanding of HME process parameters including mainly the type of extruder, and residence time distribution on protein stability with a special focus on thermal protein stability and a potential process-induced loss in lysozyme activity. The 1D and 3D simulation software Ludovic® and Ansys Polyflow®, respectively proved to be valuable tools for the evaluation of extrusion process design and provided important insights into extrusion processing optimization and potential scale-up challenges at a small-scale level as critical process steps and locations of thermomechanical stress hot spots along the process were identified. This approach supports also a rationale to identify appropriate extrusion process conditions for the production of highly protein-loaded extrudates and to define in perspective the best protein-polymer compositions. In particular, ram extrusion was identified as favored method for the production of stable protein-loaded extrudates, since the thermomechanical stress was low and still a homogenous distribution of protein particles over the cross section was achieved. However, so far, no simple 1D simulation model is available for ram extrusion thus, 3D simulations had to be employed for crucial process steps or areas, respectively. As the die in the ram extrusion was identified as the most critical process area, this limitation is negligible. In contrast, TSE strongly profits from both 1D and 3D simulations where 1D identifies critical process steps while 3D simulations boost the mechanistic understanding. Nonetheless simulation via Ludovic® is expected to better converge with experimental data when using higher feed-rates and larger size 9-, 11-, or 12-mm TSE with better instrumentation spread over the entire extrusion process (e.g., multiple temperature, die pressure, and torque monitoring). The present procedure enables a good starting point for ram extrusion and TSE trials for the production of highly-loaded protein extrudates with sufficient protein stability, protein recovery rates and homogenous distributed protein particles.

## Declaration Competing Interest

The authors declare no competing interests.

## CRediT authorship contribution statement

**Katharina Dauer:** Conceptualization, Methodology, Investigation, Data curation, Resources, Writing – original draft, Writing – review & editing, Visualization. **Felix Ellwanger:** Methodology, Investigation, Data curation, Resources, Writing – review & editing, Visualization. **Achim Overbeck:** Methodology, Investigation, Data curation, Resources, Writing – review & editing, Visualization. **Arno Kwade:** Supervision. **Heike P. Karbstein:** Supervision. **Karl G. Wagner:** Conceptualization, Resources, Writing – review & editing, Supervision.

## Declaration of Competing Interest

None.

## Data Availability

Data will be made available on request.

## Appendix A Supplementary data

Supplementary material Image 1
