# Supplementary material for: Highly protein-loaded melt extrudates produced by small-scale ram and twin-screw extrusion - evaluation of extrusion process design on protein stability by experimental and numerical approaches
Source: Int J Pharm X. 2023 Jul 1;6:100196. doi: 10.1016/j.ijpx.2023.100196 (PMC10336796; doi:10.1016/j.ijpx.2023.100196)
Supplement: Supplementary file 1 — Supplementary material [file mmc1.docx]

Supplementary Material

Highly protein-loaded melt extrudates produced by small-scale ram and twin-screw extrusion - evaluation of extrusion process design on protein integrity by experimental and numerical approaches

Katharina Dauer^1^, Kevin Kayser^1^, Felix Ellwanger^2^, Achim Overbeck^3^, Arno Kwade^3^, Heike P. Karbstein^2^, M. Azad Emin^2,4^, Karl G. Wagner^1,*^

*^1^ Department of Pharmaceutics, Institute of Pharmacy, University of Bonn, Bonn, Germany*

*^2^ Institute of Process Engineering in Life Sciences, Food Process Engineering, Karlsruhe Institute of Technology, Karlsruhe, Germany*

*^3^ Technische Universität Braunschweig, Institute for Particle Technology (iPAT), Braunschweig, Germany*

*^4^ nexnoa GmbH, Aachener Straße 1042, Köln, Germany*


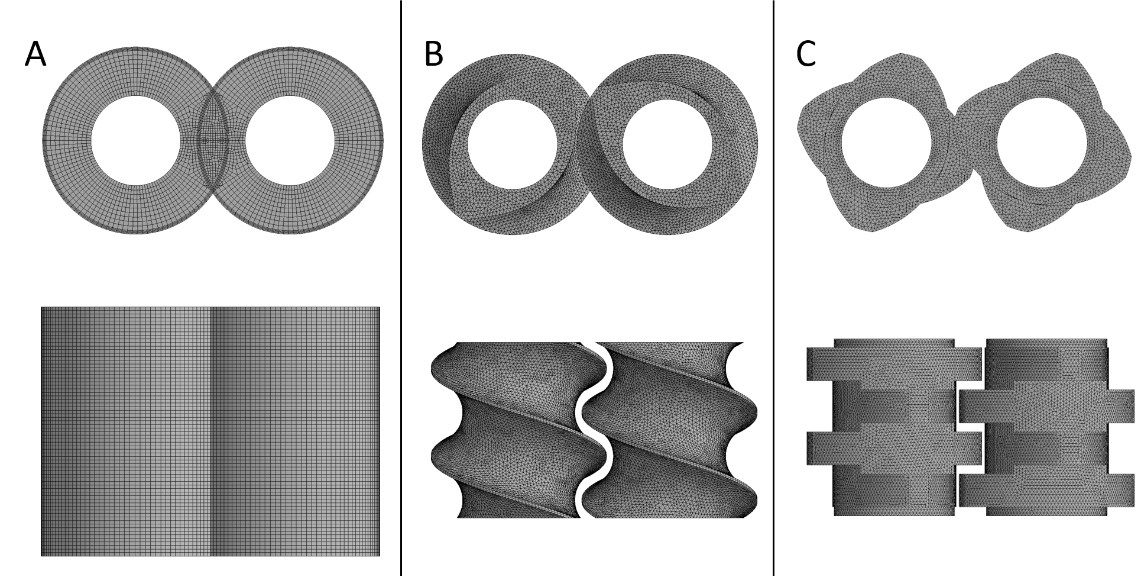


**Figure S1** Geometry and computational mesh of the TSE for **A** barrel **B** 5 mm pitch conveying elements and **C** 90° kneading elements.


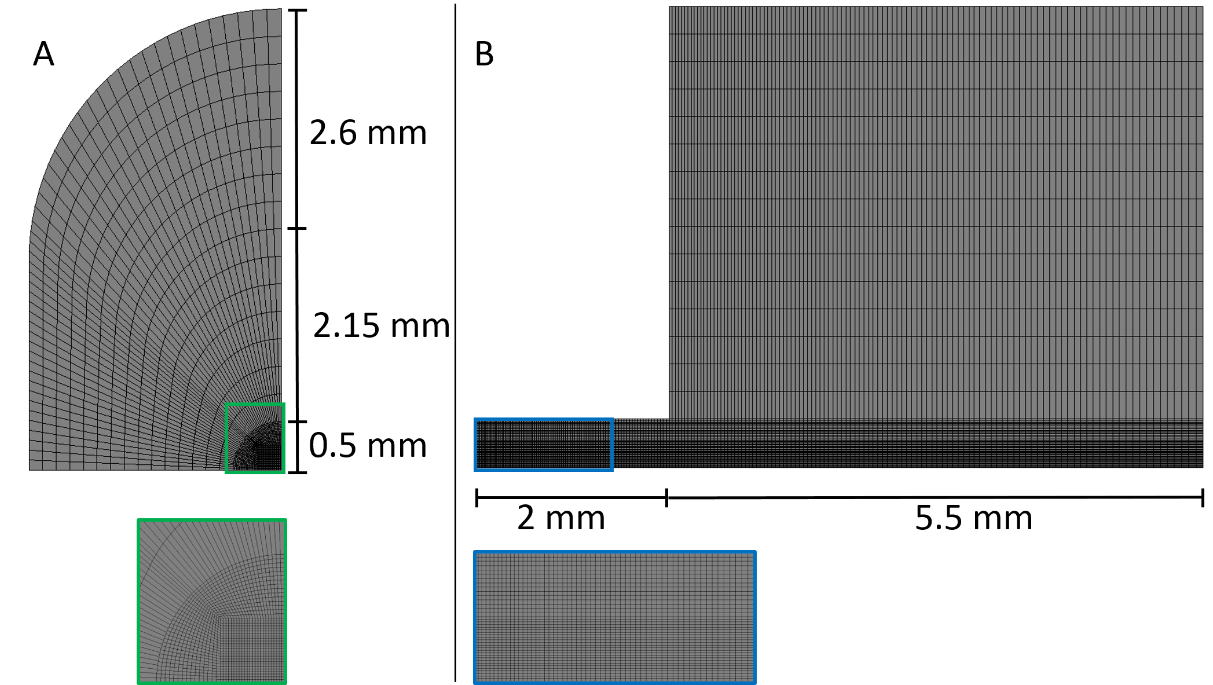


**Figure S2** Geometry and computational mesh of the TSE die for **A** front view and **B** side view of a plane of symmetry.


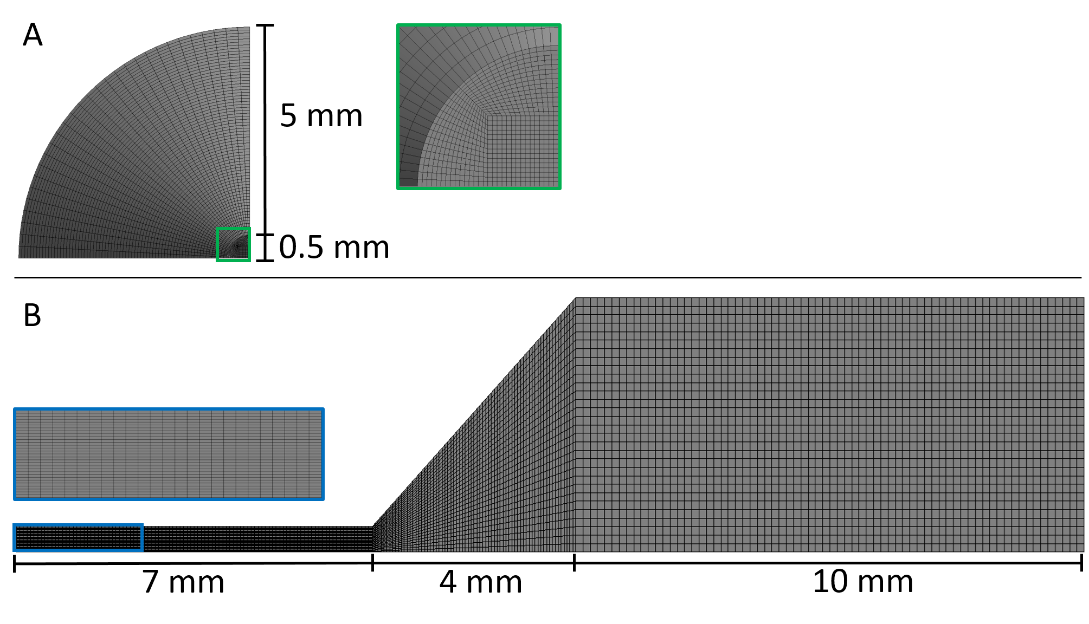


**Figure S3** Geometry and computational mesh of the ram extruder die for **A** front view and **B** side view of a plane of symmetry.

Figure S4 Comparison of the activation energy of flow of plain PEG 20,000 and mixtures containing BSA.

Figure S5 Comparison of power law index derived from utilizing the power law fit for the respective master curves of plain PEG 20,000 and mixtures containing BSA. The master curves were employed with a reference temperature of 64.0°C.

Figure S6 Comparison of the consistency derived from utilizing the power law fit for the respective master curves of plain PEG 20,000 and mixtures containing BSA. The master curves were employed with a reference temperature of 64.0°C.


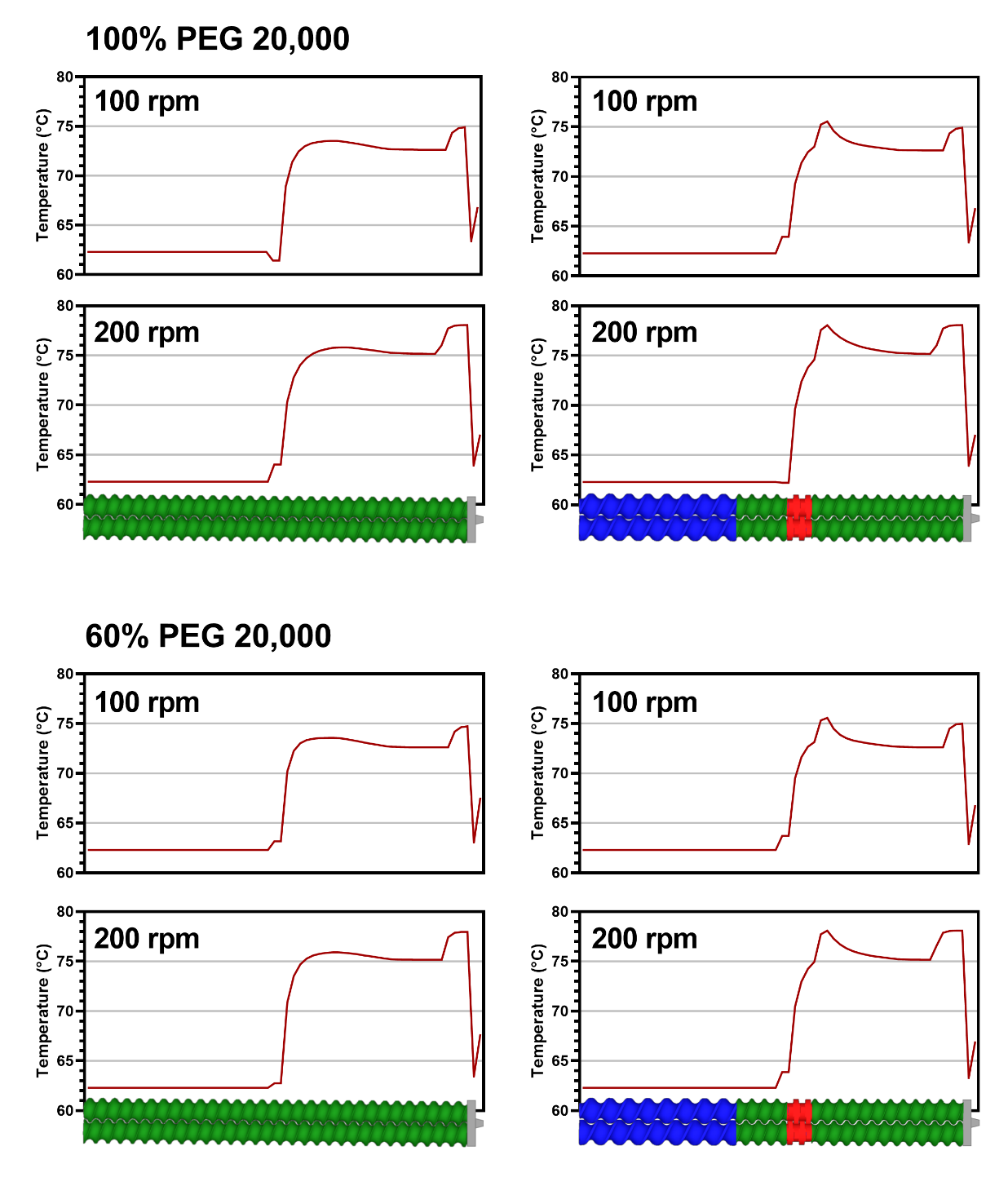


Figure S7 Exemplary presentation of simulated melt temperature of simulations based on input parameters along the extrusion process. The example shows the simulation of 100% PEG 20,000 (top) and 60% PEG 20,000/ 40% BSA (bottom) with conveying screw configuration (left) and screws containing a single 90° kneading element (red) (right) at 100 and 200 rpm screw speed with a feed-rate of 0.6 g/min.
